# Association of epidural analgesia during labor and early postpartum urinary incontinence among women delivered vaginally: a propensity score matched retrospective cohort study

**DOI:** 10.1186/s12884-023-05952-4

**Published:** 2023-09-16

**Authors:** Chuangchuang Xu, Xianjing Wang, Xiaolei Chi, Yiyao Chen, Lei Chu, Xinliang Chen

**Affiliations:** 1 Department of Obstetrics and Gynecology, International Peace Maternity and Child Health Hospital, Shanghai Jiao Tong University School of Medicine, Shanghai, China; 2 Shanghai Key Laboratory of Embryo Original Disease, Shanghai, China; 3 Shanghai Municipal Key Clinical Specialty, Shanghai, China

**Keywords:** Epidural analgesia, Urinary incontinence, Postpartum period, Primiparous women, Vaginal delivery

## Abstract

**Background:**

Although epidural analgesia is considered the gold standard for pain relief during labor and is safe for maternity and fetus, the association between the epidural analgesia and pelvic floor disorders remains unclear. Thus we estimate the association between epidural analgesia and early postpartum urinary incontinence (UI).

**Methods:**

A propensity score-matched retrospective cohort study was conducted at a university-affiliated hospital in Shanghai, China. Primiparous women with term, singleton, and vaginal delivery between December 2020 and February 2022 were included. UI was self-reported by maternity at 42 to 60 days postpartum and was classified by International Consultation on Incontinence Questionnaire-Urinary Incontinence Short Form (ICIQ-UI SF). Using logistic regression models, the associations between epidural analgesia and early postpartum UI were assessed.

**Results:**

Among 5190 participants, 3709 (71.5%) choose epidural anesthesia during labor. Analysis of the propensity-matched cohort (including 1447 maternal pairs) showed epidural anesthesia during labor was independently associated with UI in early postpartum period (aOR 1.50, 95% CI 1.24–1.81). This association was mainly contributed to stress UI (aOR 1.38, 95% CI 1.12–1.71) rather than urge UI (aOR 1.45, 95% CI 0.99–2.15) and mixed UI (aOR 1.52, 95% CI 0.95–2.45). Furthermore, we observed that the association between epidural anesthesia and UI was more pronounced among older women (≥ 35 y) and women with macrosomia (infant weight ≥ 4000 g), compared with their counterparts (both P for interaction < 0.01). After further analysis excluding the women with UI during pregnancy, the results remained largely consistent with the main analysis.

**Conclusions:**

The findings support that epidural anesthesia was associated with SUI in the early postpartum period.

**Supplementary Information:**

The online version contains supplementary material available at 10.1186/s12884-023-05952-4.

## Introduction

Epidural anesthesia has been shown to be effective in the relief of labor pain. According to national surveys, about 80% of women in developed countries choose epidural anesthesia during labor [1, 2]. One such proportion can still reach 50% to 75% in economically developed regions of developing countries, including Shanghai, China [3].

Several investigators have evaluated the association between epidural anesthesia and maternal–fetal outcomes, but there was no consensus on the effect of epidural anesthesia on postpartum pelvic floor function in women [4, 5]. Epidural anesthesia would be a potential risk factor for postpartum pelvic floor disorders, particularly urinary incontinence (UI), as it can extend the duration of force on pelvic floor muscles and nerves by prolonging the second stage of labor [6–10]. Paradoxically, anesthesia has a great relaxant effect, which contribute to a significant decline in pelvic floor injury during labor and further probably reduce the incidence of postpartum UI [11].

One study reported that epidural anesthesia was associated with the onset of stress UI (SUI) but not with urge UI (UUI) or mixed UI (MUI) [12]; Other studies reported no association between epidural anesthesia and UI in the early postpartum period [13–15]; Even Ruan et al. found in a 63-person cohort study that epidural anesthetic reduced PFM muscle tone in the early postpartum period, preventing the prevalence of UI [11]. However, the accurate conclusions could not be draw owing to limited sample sizes and the confounding factors such as delivery model.

Epidemiological surveys in the United States, Europe, and Asia show a prevalence of UI of approximately 30% [16–18]. In Shanghai, China, the prevalence of UI in women of childbearing age and aged women ranges from 23.3 to 36.5% [19]. Women with persistent UI had lower quality of life [20] and huge social costs burden [21]. Therefore, it is crucial to identify the risk factors for early postpartum UI. To investigate this, we examined the association between epidural anesthesia and early postpartum UI in a large propensity-matched cohort study of women with a first vaginal delivery.

## Methods

### Design, setting, and participants

This single-center retrospective cohort study was conducted at International Peace Maternity and Child Health Hospital (IPMCH) from December 2020 to February 2022. At 42–60 days postpartum, maternity appointment to the institutional Pelvic Floor Rehabilitation Center for pelvic floor related examinations. The physician will consult each woman if she suffers from symptoms related to pelvic floor disorders through a standardized questionnaire. Results were recorded into an electronic health record and matched to the maternal baseline data in the electronic medical record by a unique hospitalization number.

Only primiparous women who delivered vaginally were included to avoid confounding by number of deliveries as well as mode of delivery. The exclusion criteria were as follows: (1) Preterm births; (2) Twin births; (3) Postpartum visits beyond 42 to 60 days; (4) Without baseline data (e.g., height, weight, and labor summaries).

The protocol was approved by the Ethics Committee of IPMCH (GKLW-2023-024-01), and the requirement for individual consent was waived. The study is reported according to the Strengthening the Reporting of Observational Studies in Epidemiology (STROBE) guideline.

### Exposure: epidural analgesia

Since the cost of epidural anesthesia was covered by medical insurance in Shanghai, the choice of epidural anesthesia was determined by the individual's wishes, not by socioeconomic status. Prior to the procedure, the obstetrician and anesthesiologist jointly assessed the basic condition of the parturient women to exclude contraindications. The timing of anesthesia was chosen when the cervix was dilated 3–4 cm, and the epidural catheter was implanted in the L2-L3 intervertebral space. The rate of infusion was adjusted as necessary to maintain the labor analgesic effect without causing motor blockage. Following that, professional nursing personnel will attend to the women and notify the obstetrician if labor progress is sluggish or the fetal heartbeat is weak, and the obstetrician will determine whether to continue the labor or intervene artificially.

### Study outcomes

The primary outcome was UI, which was defined by International Urogynecological Association (IUGA) and International Continence Society (ICS) as any involuntary urine leakage [22]. Women were further assessed by a physician using the International Consultation on Incontinence Questionnaire Short Form (ICIQ-UI SF) [23] if they self-reported symptoms of urine leaking after delivery. The type of UI was determined primarily by Sect. 6 of the questionnaire. SUI was diagnosed in participants who chose "leaks when you cough or sneeze"/"leaks while you are physically active/exercising" from the list of options. UUI was diagnosed in participants who chose "leaks before you can get to the toilet"/"leaks when you are asleep"/"leaks when you have finished urinating and are dressed " from the list of options. While both symptoms were present in the participants, MUI was diagnosed. This questionnaire is now available in Chinese, and its test validity and accuracy have been well validated [24].

### Covariates and definitions

Baseline characteristic variables included maternal age, prenatal BMI (calculated from early pregnancy weight and height), pregnancy weight gain ratio (pregnancy weight gain divided by weight at the early pregnancy), abortion history, complications (hypertensive disorders; gestational diabetes/pre-pregnancy diabetes; others, defined as anemia, impaired liver and kidney function, and abnormal thyroid function), gestation week, induction of labor (oxytocin, prostaglandin, and cervix balloon mechanical induction of labor), infant weight, and infant head circumference.

Delivery characteristic variables included first stage of labor (time from regular uterine contractions to cervix fully dilated), second stage of labor (time from cervix full dilated to complete delivery of fetus), presence of perineal lacerations, use of episiotomy, and use of instrumental birth.

### Statistical analysis

The frequency (percentage) of categorical variables and the median (interquartile range) or mean (standard deviation) of continuous variables were used to report descriptive statistics. Propensity-matched scoring was applied to achieve the balance of baseline data in the exposed and control groups (i.e., minimal confounding). A multivariate logistic regression model was used to calculate the propensity score, with epidural anesthesia as the dependent variable. Age, prenatal BMI, pregnancy weight gain ratio, abortion history, complications, gestation week, infant weight, and infant head circumference were the covariates factors. The caliper width was set at 0.02 and the matching procedure was completed with a 1:1 ratio and no replacement (greedy matching method). The absolute standardized mean difference (SMD) was used to estimate the balance of baseline data between the two groups before and after matching. SMD value lower than 0.1 was considered a good balance. Distribution of propensity scores in the sFigure 1 of supplementary material.

In the matched cohort, odds ratio (OR) and their 95% confidence interval (CI) of outcomes were estimated for women with and without epidural analgesia use. Previous studies have reported stage of labor, perineal lacerations, episiotomy, and instrumental birth as potential risk factors for the development of postpartum UI. Considering that these factors could not be classified as baseline characteristics, we included them in a binary logistic regression model to obtain an adjusted ORs in the post-matched cohort.

Two sensitivity analyses were undertaken. First, considering that age, prenatal BMI, pregnancy weight gain ratio, infant weight and infant head circumference related to postpartum UI, and that epidural anesthesia prevalence vary according to the rate of induction of labor, we stratified participants according to age (< 35 years, ≥ 35 years), prenatal BMI (< 25 kg/m2, ≥ 25 kg/m2), pregnancy weight gain ratio (< 20%, ≥ 20%), infant weight (< 4000 g, ≥ 4000 g), infant head circumference (< 34 cm, ≥ 34 cm), and induction of labor (no, yes). Second, considering possible confounding causality, women with UI during pregnancy may already have abnormal pelvic floor function and are more likely to report symptoms of UI after delivery. We analyzed whether the association would change if only individuals who had UI at postpartum were selected.

Statistical and graphing software were done with R version 4.1.3. All statistics were two-sided tests, and P < 0.05 was considered statistically significant.

## Results

### Characteristics of the participants

**Fig. 1:**
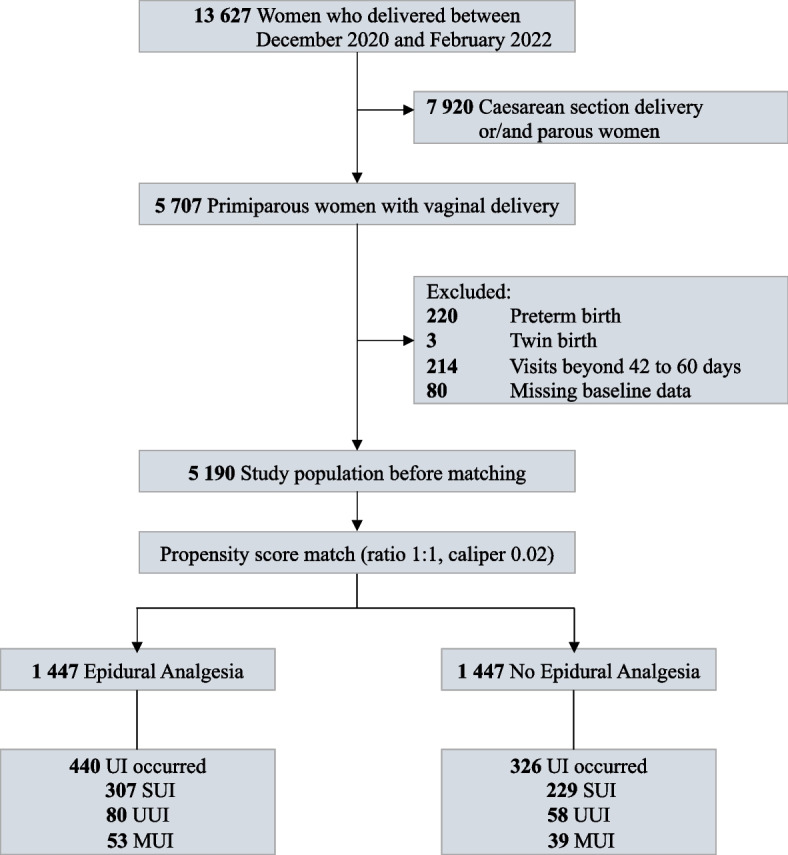
Identification of eligible participants for cohort. Abbreviation: UI, urinary incontinence; SUI, stress urinary incontinence; UUI, urge urinary incontinence; MUI, mixed urinary incontinence

A total of 13 627 women were delivered in the IPMCH from December 2020 to February 2022, and 8 437 women were excluded as follows: 7 920 were delivered via cesarean section or/and parity ≥ 2; 220 were preterm delivery; 3 were twin births; 214 had a visits beyond 42 to 60 days postpartum; and 80 had missing baseline data. The final 5 190 participants were enrolled in the study, of which 3 709 had epidural anesthesia at delivery. The propensity score-matched cohort included 2 894 primiparous women, 1 447 each in the epidural and non-epidural groups (Fig. 1).

The former part of Table 1 describes the baseline characteristics of participants. Before propensity matching, women who underwent epidural analgesia had a higher gestation week (39 [38, 40] vs. 39 [38, 40], *P* < 0.001, SMD = 0.205), a higher infant weight (3317.00 [343.39] vs. 3222.13 [349.62], *P* < 0.001, SMD = 0.274), and a higher incidence of induced labor (1614 [43.5] vs. 393 [26.5], *P* < 0.001, SMD = 0.362). After propensity matching, a total of 1 447 individual pairs of study participants were matched and successfully entered the final cohort, with well-balanced baseline data (SMD less than 0.1).

**Table 1:** Baseline and delivery characteristics of participant before and after propensity score matching

	Pre-matching, Epidural Analgesia				Post-matching, Epidural Analgesia			
Characteristics	No (*N* = 1481)	Yes (*N* = 3709)	*P*-value	SMD	No (*N* = 1447)	Yes (*N* = 1447)	*P*-value	SMD
**Baseline characteristics**								
Age, mean (SD), y	29.89 (3.19)	29.85 (3.10)	0.607	0.016	29.90 (3.21)	29.89 (3.08)	0.943	0.003
Pre-pregnancy BMI, mean (SD), kg/m^2^	22.71 (3.68)	23.32 (29.89)	0.429	0.029	22.73 (3.71)	22.70 (2.53)	0.804	0.009
Weight gain in pregnancy, median [IQR], kg	12 [9, 15]	12 [9, 15]	0.576	0.017	12 [9, 15]	12 [10, 15]	0.689	0.015
Abortion history (%)	422 (28.5)	1017 (27.4)	0.455	0.024	408 (28.2)	427 (29.5)	0.460	0.029
Complications								
Hypertensive disorders (%)	75 (5.1)	170 (4.6)	0.506	0.022	72 (5.0)	71 (4.9)	1.000	0.003
GDM/PGDM (%)	182 (12.3)	516 (13.9)	0.133	0.048	181 (12.5)	181 (12.5)	1.000	< 0.001
others (%)	175 (11.8)	516 (13.9)	0.050	0.063	172 (11.9)	179 (12.4)	0.733	0.015
Gestation week, media [IQR], w	39 [38, 40]	39 [38, 40]	< 0.001	0.205	39 [38, 40]	39 [38, 40]	0.330	0.031
Infant weight, mean (SD), g	3222.13 (349.62)	3317.00 (343.39)	< 0.001	0.274	3238.07 (336.47)	3240.64 (338.07)	0.838	0.008
Infant head circumference, median [IQR], cm	34 [33, 35]	34 [33, 35]	0.343	0.029	34 [33, 35]	34.00 [33, 35]	0.672	0.016
Induction of labor (%)	393 (26.5)	1614 (43.5)	< 0.001	0.362	393 (27.2)	398 (27.5)	0.867	0.008
**Delivery characteristics**								
First stage of labor, median [IQR], min	240 [180, 360]	465 [330, 600]	< 0.001	NA	240 [180, 360]	450 [330, 600]	< 0.001	NA
Second stage of labor, median [IQR], min	32 [19, 55]	46 [30, 72]	< 0.001	NA	32.00 [20, 55]	45 [30, 68]	< 0.001	NA
Instrumental birth (%)	196 (13.2)	474 (12.8)	0.693	NA	194 (13.4)	194 (13.4)	1.000	NA
Perineal lacerations (%)	1021 (68.9)	2673 (72.1)	0.027	NA	992 (68.6)	1061 (73.3)	0.005	NA
Episiotomy (%)	418 (28.2)	984 (26.5)	0.228	NA	412 (28.5)	365 (25.2)	0.054	NA

*Abbreviation*: *BMI* Body mass index, *IQR* Interquartile range, *NA* Not applicable, *SMD* Standardized mean difference

The latter part of Table 1 describes the delivery characteristics of the participants. In the propensity-matched cohort, women with epidural anesthesia during labor had a longer first (450 [330, 600] vs. 240 [180, 360], *P* < 0.001) and second (45 [30, 68] vs. 32 [20, 55], *P* < 0.001) stages of labor. The rate of perineal laceration was higher in the epidural group compared to the non-epidural group (2258 [73.6] vs. 2119 [69.1], *P* = 0.005), but no statistically significant differences were found in the incidence of instrument birth (194 [13.4] vs. 194 [13.4], *P* = 1.000) and episiotomy (365 [25.2] vs. 412 [28.5], *P* = 0.054) between the two groups.

### Association of epidural anesthesia and early postpartum UI

In the propensity-matched cohort, the incidence of UI was higher in the epidural analgesia group than in the no epidural analgesia group (440 [30.4] vs. 326 [22.5]; adjusted OR 1.50, 95% CI 1.24–1.81). Further studies of each subtype of UI found similar results in SUI (307 [21.2] vs. 229 [15.8]; adjusted OR, 1.38 [95% CI, 1.12–1.71]). The difference of the incidence of UUI (80 [5.5] vs. 58 [4.0]; adjusted OR, 1.45 [95% CI, 0.99–2.15]) and MUI (53 [3.7] vs. 39 [2.7]; adjusted OR, 1.52 [95% CI, 0.95–2.45]) between the two groups was not statistically significant (Table 2).

**Table 2:** Incidence of outcomes and ORs in propensity score matched cohort

Outcomes	Event, No. (%)	Unadjusted OR (95% CI)	*P*-value	Adjusted OR (95% CI)^a^	*P*-value
UI					
Epidural analgesia	440 (30.4)	1.50 (1.27–1.78)	< 0.001	1.50 (1.24–1.81)	< 0.001
No epidural analgesia	326 (22.5)	1 [Reference]		1 [Reference]	
SUI					
Epidural analgesia	307 (21.2)	1.43 (1.19–1.73)	< 0.001	1.38 (1.12–1.71)	0.003
No epidural analgesia	229 (15.8)	1 [Reference]		1 [Reference]	
UUI					
Epidural analgesia	80 (5.5)	1.40 (0.99–1.99)	0.056	1.45 (0.99–2.15)	0.059
No epidural analgesia	58 (4.0)	1 [Reference]		1 [Reference]	
MUI					
Epidural analgesia	53 (3.7)	1.37 (0.90–2.10)	0.139	1.52 (0.95–2.45)	0.082
No epidural analgesia	39 (2.7)	1 [Reference]		1 [Reference]	

*Abbreviation*: *UI* Urinary incontinence, *SUI* Stress urinary incontinence, *UUI* Urge urinary incontinence, *MUI* Mixed urinary incontinence, *OR* Odds ratio, *CI* Confidence interval

^a^Adjusted for first stage of labor, second stage of labor, instrumental birth, episiotomy, and perineal lacerations

### Sensitivity analysis

**Fig. 2:**
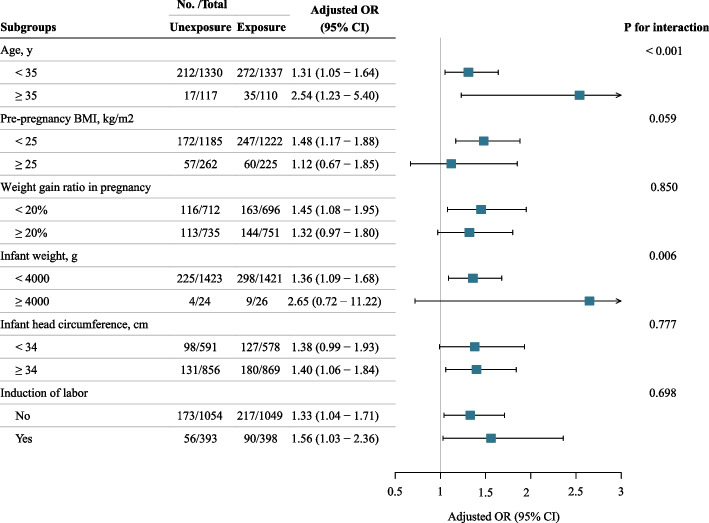
Association of epidural analgesia with early postpartum SUI in subgroup analyses. BMI, body mass index; SUI, stress urinary incontinence; OR, odds ratio; CI, confidence interval. ORs and 95% CIs were calculated using unexposed group (no epidural analgesia use) as reference, and adjusted for first stage of labor, second stage of labor, instrumental birth, episiotomy, and perineal lacerations

For each subgroup, those who received epidural analgesia were matched with those who did not before the sensitivity analysis. The propensity score-matched group had well-balanced covariates with no statistically significant differences. We observed significantly interactions between age (P for interaction < 0.001) and infant weight (P for interaction = 0.006) on the relationship between epidural analgesia and early postpartum SUI. The association between the epidural analgesia and early postpartum SUI was more pronounced among older women (> 35 y) and women with macrosomia (infant weight ≥ 4000 g), compared with their counterparts (Fig. 2).

**Fig. 3:**
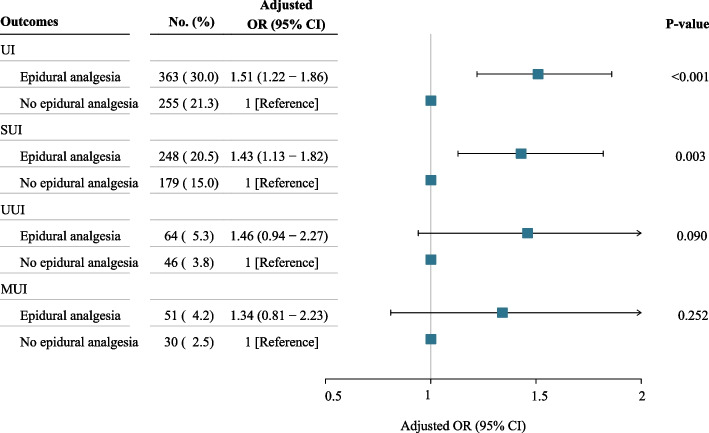
Association between epidural analgesia and outcomes excluding participants whose UI during pregnancy. UI, urinary incontinence; SUI, stress urinary incontinence; UUI, urge urinary incontinence; MUI, mixed urinary incontinence; OR, odds ratio; CI, confidence interval. ORs and 95% CIs were adjusted for first stage of labor, second stage of labor, instrumental birth, episiotomy and perineal lacerations

We reanalyzed our data after excluding participants who had UI during pregnancy. The levels of ORs for UI and different subtypes of UI between the two groups were comparable to the overall participants (Fig. 3).

## Discussion

In this propensity score-matched cohort study using data from 5190 primiparous women, our findings confirmed epidural anesthesia during labor was independently associated with UI in early postpartum period. When compared to the no epidural anesthesia group, the odds of having UI were higher in the epidural anesthesia group after adjustment for potential confounding factors. This association was mainly explained by the increased prevalence of SUI rather than UUI and MUI. Furthermore, we observed that the strength of the association between epidural anesthesia and postpartum SUI varied when stratified the participants according to age and infant weight. After further analysis excluding the women with UI during pregnancy, the results remained largely consistent with the main analysis. In addition, we found that epidural anesthesia was associated with a prolonged first and second stages of labor, and increased the incidence of perineal laceration.

The longitudinal cohort studies show that approximately two-thirds women who had early postpartum UI would develop persistent UI after 12 years [20]. Therefore, considering the long-term adverse impact of postpartum UI, it is necessary to promptly provide early identification and rehabilitation exercises for women. To the authors' knowledge, there is a lack of randomized controlled trials that have studied the association of epidural anesthesia with different subtypes of UI. Wang et al. reported that UI at 6 weeks postpartum was not associated with epidural anesthesia in a retrospectively corhort study of 333 women [15]. However, only 19% of participants in this study received epidural anesthesia during labor, limiting the power. Our findings were consistent with the results of Rortveit et al. [12] indicating that, in comparison with no epidural anesthesia, women who received epidural anesthesia during labor did show an increased risk of UI.

Despite years of etiologic research, the pathogenesis of UI remains unclear. The main feature of SUI is predictable urine loss during activities that increase intra-abdominal pressure (e.g., exercise, laughing, sneezing) [25], compared to UUI, which is characterized by involuntary urine loss with urgency as well as increased urinary frequency or nocturia [26]. The present study suggests that epidural anesthesia is only associated with the onset of postpartum SUI. This seems to suggest that different types of UI occur through different mechanisms, which we believe needs to be further explored in the future.

We observed an interaction between age and epidural anesthesia. There was a stronger association between epidural anesthesia and odds of having postpartum SUI in older women (≥ 35 y) compared with those younger women (< 35 y). Aging has been reported to be associated with the prevalence of UI [27, 28], and that the effects of exercise or natural recovery from UI may be more pronounced in younger women than in older women [29]. Furthermore, a long-term longitudinal cohort study showed that the difference in the incidence of postpartum UI due to mode of delivery was not statistically significant in the 40-year-old population [30]. This may be due to the progressive dominance of aging in the pathogenesis of UI with advancing age.

We observed a stronger association between epidural anesthesia and postpartum SUI in women with infant weight ≥ 4000g, compared to women with infant weight < 4000 g. Previous studies have shown that higher infant weight leads to greater pelvic floor stress during labor, which induces abnormal bladder and urethral positioning and further contributes to UI [31–33]. We hypothesize that higher infant weight and epidural anesthesia have a synergistic effect on onset of postpartum UI. However, the intrinsic connection still needs further investigation.

Epidural anesthesia was significantly associated with a prolonged stage of labor in the current study, which confirms the previously common views [34, 35]. Interestingly, epidural anesthesia did not increase the risk of instrumental birth and episiotomy, but increased the risk of perineal lacerations, which was different from the findings of previous studies [36]. We remain cautious about this last finding, as it has been a topic of great discussion. In summary, after adjusting for the confounding factors mentioned previously, we found that epidural anesthesia was an independent risk factor for early postpartum UI after.

### Limitations

This study had a large sample size, but it still had several limitations. First, the current study is a retrospective study, which has an inherent selection bias and some important variables (e.g., the intensity of postpartum physical activity, and breastfeeding or not in the postpartum period) cannot be collected. Second, urodynamic parameters such as bladder urine residue, urinary flow rate, and other indicators were not acquired. Due to the considerable medical costs involved, these were not used as standard postpartum examinations. Despite the presence of professionals on hand to guide patients, the use of questionnaire scales to determine the diagnosis of urinary incontinence and its type is still subjective, especially since a lot of times there is an under-reporting of the condition due to inherent shame by patients. Third, this study was limited to the early postpartum period, and further investigation is needed regarding changes in the incidence of UI over time and the association between early and persistent UI. Finally, almost all of the participants in this study were residents of Shanghai, China, an economically more developed region of China. Thus, our results may not be generalizable to all Chinese people. Further studies need to be conducted to replicate our findings in a nationally representative sample.

## Conclusions

Among primiparous women who underwent vaginal delivery, the use of epidural anesthesia during labor was significantly associated with the prevalence of early postpartum SUI. Screening for early postpartum urinary symptoms should be emphasized for women who have undergone epidural anesthesia during delivery.

### Supplementary Information

**Additional file 1: sFigure1.** Distribution of propensity scores before and after propensity scoring match.

## Data Availability

The data analyzed during the study is available from the corresponding author upon reasonable request.

## Abbreviations

UI: Urinary incontinence
SUI: Stress urinary incontinence
UUI: Urge urinary incontinence
MUI: Mixed urinary incontinence
ICIQ-UI SF: International Consultation on Incontinence Questionnaire-Urinary Incontinence Short Form
IUGA: International Urogynecological Association
ICS: International Continence Society
IPMCH: International Peace Maternity and Child Health Hospital
BMI: Body mass index
SMD: Standardized mean difference
OR: Odds ratio
CI: Confidence interval
