# Supplementary material for: Association of epidural analgesia during labor and early postpartum urinary incontinence among women delivered vaginally: a propensity score matched retrospective cohort study
Source: BMC Pregnancy Childbirth. 2023 Sep 16;23:666. doi: 10.1186/s12884-023-05952-4 (PMC10504782; doi:10.1186/s12884-023-05952-4)
Supplement: Supplementary file 1 — Additional file 1: sFigure1. Distribution of propensity scores before and after propensity scoring match. [file 12884_2023_5952_MOESM1_ESM.docx]

sFigure1 Distribution of propensity scores before and after propensity scoring match.


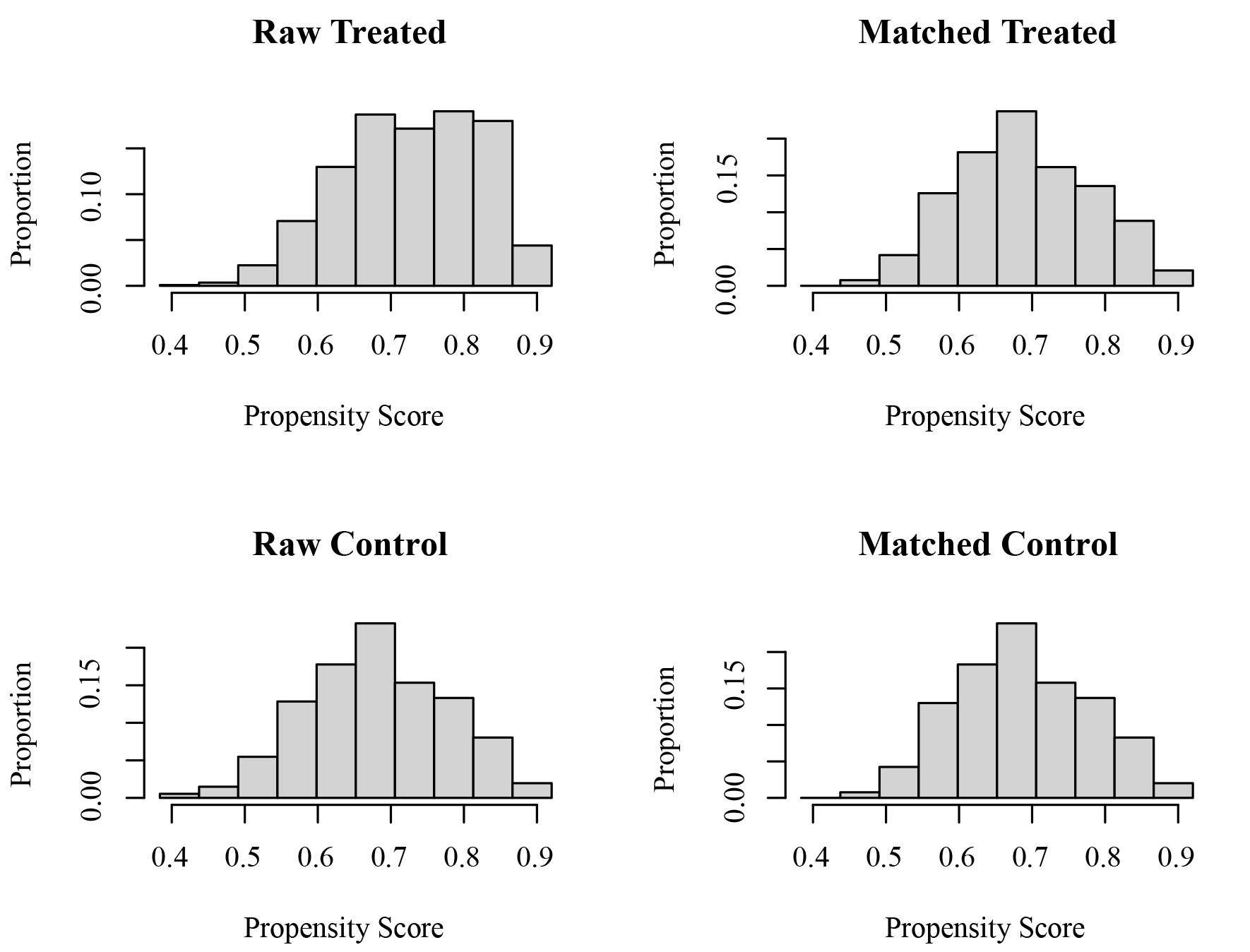


(a)


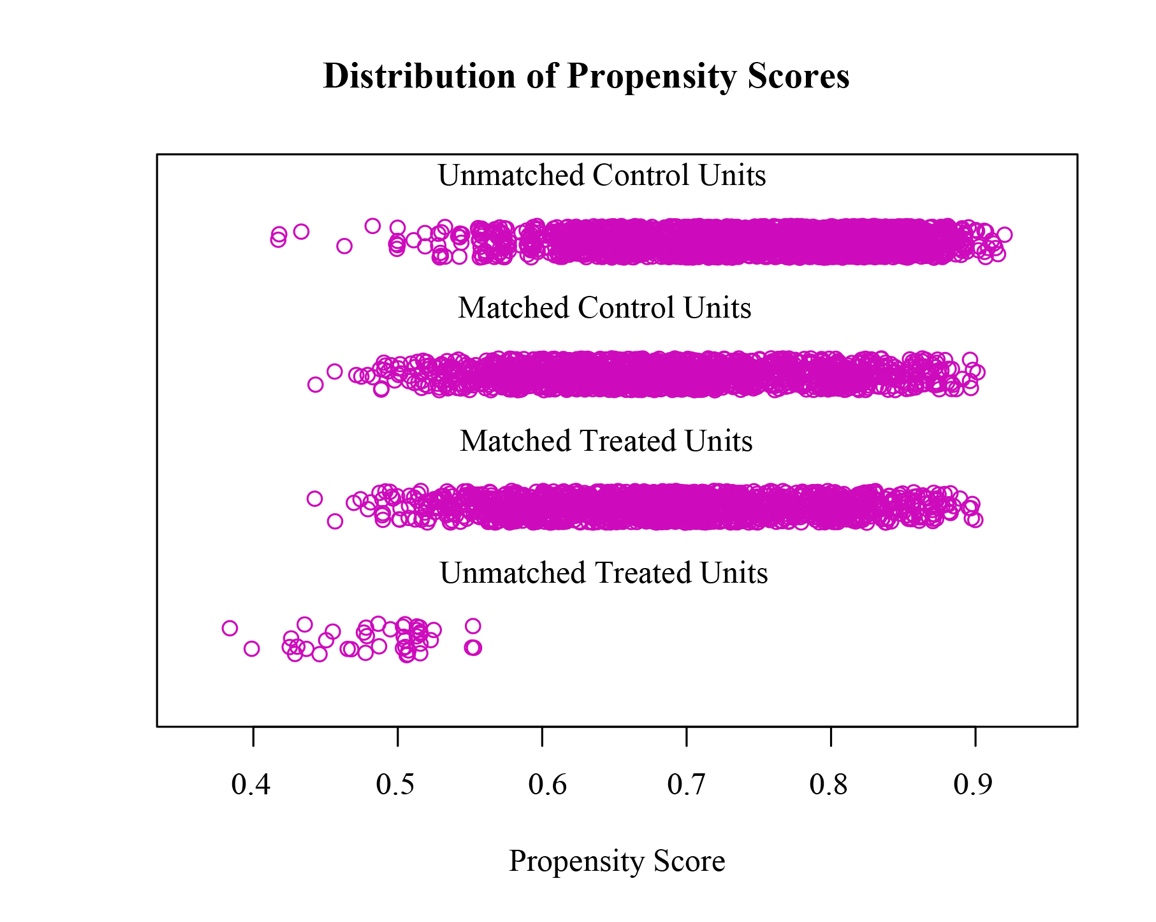


(b)

Treated Units indicates no-epidural group; Control Units indicates epidural group.
